# Optimization of the recombinant production and purification of a self-assembling peptide in *Escherichia coli*

**DOI:** 10.1186/s12934-014-0178-0

**Published:** 2014-12-31

**Authors:** Mazda Rad-Malekshahi, Matthias Flement, Wim E Hennink, Enrico Mastrobattista

**Affiliations:** Department of Pharmaceutics, Utrecht Institute for Pharmaceutical Sciences (UIPS), Utrecht University, 3584 CG Utrecht, Netherlands; Department of Pharmaceutics, Faculty of Pharmacy, Tehran University of Medical Sciences, Tehran, Iran

**Keywords:** Self-assembly, Amphiphilic peptide, SUMO protein, Recombinant expression, Selective precipitation

## Abstract

**Background:**

Amphiphilic peptides are important building blocks to generate nanostructured biomaterials for drug delivery and tissue engineering applications. We have shown that the self-assembling peptide SA2 (Ac-AAVVLLLWEE) can be recombinantly produced in *E. coli* when fused to the small ubiquitin-like modifier (SUMO) protein. Although this system yielded peptides of high purity with no residual amino acids after cleavage of the SUMO fusion protein, the yield after purification was generally low (~1 mg/L bacterial culture) as compared to other peptides and proteins produced with the same method and under the same conditions.

**Results:**

The aim of this study is to understand the underlying mechanisms causing the low yield of this recombinant peptide in *E. coli* and to optimize both production and purification of recombinant SA2 peptides. It was demonstrated that by simply changing the medium to a well-balanced auto-induction medium the yield of recombinant production was augmented (~4 fold). Moreover, it was demonstrated that self-assembly of SUMO-SA2 fusion proteins caused the low peptide yields after purification. By replacing the second IMAC purification step with a selective precipitation step, peptide yields could be increased approx. 3 fold. With these optimizations in place the overall yield of purified SA2 peptide increased with 12-fold.

**Conclusion:**

Premature self-assembly of the SUMO-SA2 fusion construct interfered with proper purification of the SA2 peptide, resulting in low yields of purified peptide and this could be prevented by changing the mode of purification. These findings are important when setting up purification schemes for other self-assembling peptides with the use of a SUMO fusion construct.

## Introduction

Amphiphilic peptides (Aps) represent a group of small peptides with sequestered hydrophobic and hydrophilic domains. Their amphiphilic nature allows them to self-assemble into supramolecular structures, such as micelles, nanotubes, belts or vesicles with interesting applications in drug delivery and tissue engineering [1-10].

Aps are produced by solid-phase peptide synthesis (SPPS) [11,12] but also recombinantly in bacteria and yeast [13-16]. Production of Aps via SPPS has some limitations. The presence of large stretches of hydrophobic amino acids in Aps may cause collapse of the peptides on the solid support, which increases the risk of truncated peptides that even become more problematic in large scale synthesis [17]. Such truncated peptides which often differ in only one amino acid from the full length peptide, are difficult to remove during subsequent purification steps. It was shown that such impurities can have profound effects on the self-assembling behavior of these Aps [17]. Besides purity, high scale production of SPPS is costly [18,19].

Numerous attempts have been made for recombinant production of relatively small, self-assembling peptides. However, such methods have encountered several challenges: their amphiphilic nature and tendency to self-assemble can cause toxicity problems in the production cells or can lead to proteolytic degradation or sequestering in inclusion bodies [20]. To prevent this, APs are often fused to a larger chaperone proteins [21].

**Figure 1:**
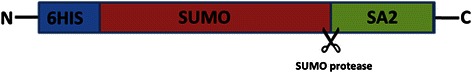
**Schematic illustration of 6His-tagged SUMO-SA2.** SUMO protease can specifically cut the recombinant construct and release the SA2 peptide.

One such fusion construct that favors soluble production of small hydrophobic peptides is the small ubiquitin-like modifying protein (SUMO) [21]. It can protect the protein/peptide by using its chaperoning properties, enhance the solubility and increase production [20]. This relatively small fusion protein (12.2 KD) can be specifically cleaved and separated from its fused partner by SUMO protease [22]. An important advantage of the SUMO fusion protein technology is that it generates peptides with a native N-terminus without residual amino acids after cleavage with SUMO protease [22,23]. In previous studies, we have used the SUMO fusion technology for the recombinant production and purification of a 10 amino-acid amphiphilic peptide called SA2 (AAVVLLLWEE) in *E. coli* [6]. The fusion protein consisted of a 6 residue histidine tag at the N-terminus for purification, SUMO for stability and solubility and the SA2 peptide at the C-terminus. Cleavage of the fusion protein with SUMO protease enabled the release of SA2 without any residual amino acids (Figure 1). Since SA2 self-assembles into nanovesicles, the SUMO fusion technology was used to prevent premature self-assembly of the peptides into supramolecular structures and to keep the monomeric peptide soluble during recombinant production. Although we succeeded to purify SA2 peptides following this approach, the yield was rather low, with approximately 1 mg of purified SA2 peptide/L bacterial culture.

In this study, the aim was therefore to determine and optimize the critical steps in the production and purification scheme of SA2 that limits the purified peptide yield and to maximize production yields of SA2 peptide.

## Results and discussion

### Peptide biosynthesis

To increase the yield of purified recombinant SA2, we first focused on the optimization of production of SUMO-SA2 in *E. coli.* Production of potentially toxic proteins generally does not benefit from the use of strong promoters to maximize transcription levels. Instead, tight control over the induction of expression is necessary to prevent premature expression and subsequent toxicity to the host. Strategies that enable induction of expression at high cell densities using auto-induction medium have been used to yield good levels of recombinant proteins [24]. Moreover, auto-induction media have the added advantage of very low to no expression prior to the time of induction because of the catabolyte repression effect of glucose, which makes it particularly suitable for the expression of potentially toxic recombinant proteins [25]. Here, we tested two different media for recombinant production of SA2: 1). Standard LB medium with IPTG as irreversible inducer of SUMO-SA2 expression under control of the *T7lac* promoter, and 2). ZYM medium, which leads to auto-induction of SUMO-SA2 expression based on glucose as preferable carbon source for *E. coli*. A restricted concentration of glucose not only is consumed preferentially during growth but also inhibits uptake of lactose. After consumption and depletion of glucose to reach a high cell density, lactose will be taken up and converted to the allolactose which is an inducer for T7 RNA polymerase expression under control of the *lacUV5* promoter and unblocks the *T7lac* promoter, leading to high levels of expression [24]. A 5 ml overnight culture of *E. coli* was diluted in 1000 ml of LB or ZYM medium followed by incubation at 37°C in a shaking incubator. OD_600_ was monitored for LB medium and IPTG was added when the OD_600_ was ~0.4. Four hrs after induction with IPTG (in case of LB medium) and 16 hrs after inoculation (in case of ZYM medium) the wet weight of the bacterial pellet was determined (Table 1). A higher biomass (~3.5 fold) was reached using the ZYM autoinduction medium in comparison with IPTG induction in LB medium [24].

**Table 1:** **Yield of biomass, SUMO-SA2 and SA2 (mg) produced per liter of LB or ZYM medium**

**Medium**	**Wet Biomass (g)**	**SUMO-SA2 (mg)**	**Expected amount of SA2 (mg)**	**Amount of cleaved SA2 (mg)**	**Amount of purified SA2 (mg)**
LB + 1 mM IPTG	2.18 ± 0.11	63 ± 2	5.04 ± 0.18	4.26 ± 0.15	3.20 ± 0.20
ZYM (Auto induction)	7.62 ± 0.03	243 ± 5	19.44 ± 0.45	16.21 ± 0.32	12.16 ± 0.43

These results show that compared to induction with IPTG, the auto induction medium significantly increased SUMO-SA2 production and subsequently the yield of purified SA2 peptides (>3.8 times).

### SUMO-SA2 purification

After induction, *E.coli* cells were harvested and lysed. Cleared lysate was used for further purification of the SUMO-SA2 construct using Ni^2+^-NTA immobilized metal affinity chromatography. Eluted proteins were subjected to a buffer exchange with HEPES, pH 8.0 using a Hiprep 26/10 desalting column. Determination of total amount of SUMO-SA2 after desalting based on its extinction coefficient at 280 nm (6990 M^−1^ cm^−1^) indicated higher protein yield per volume (~3.8 time) using the ZYM autoinduction medium in comparison with IPTG induction in LB medium (Table 1).

**Figure 2:**
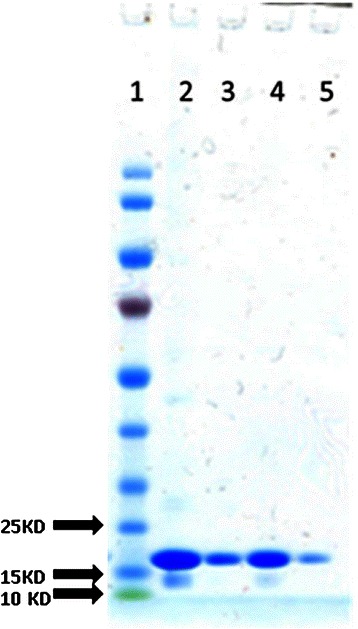
**SDS-PAGE analysis of purified SUMO-SA2.** Lane 1: 3 μl PageRuler™ Prestained Protein Ladder (Fermentas, Vilnius, Lithuania). Lane 2: 25 μl of purified SUMO-SA2 before desalting. Lane 3: same as lane 2, but 10x diluted in sample buffer. Lane 3: 25 μl of purified SUMO-SA2 after desalting. Lane 4: same as lane 3 but 10x diluted in sample buffer.

**Figure 3:**
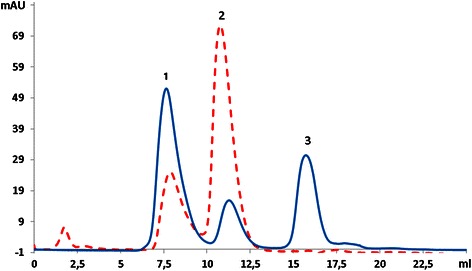
**Size exclusion chromatography of SUMO-SA2 before (dashed red line) and after (solid blue line) enzymatic cleavage with SUMO protease.** Peak 1 corresponds to protein eluting in the void volume, peak 2 corresponds to SUMO-SA2 or cleaved SUMO and peak 3 corresponds to SA2 peptide.

SDS-PAGE analysis of SUMO-SA2 before and after desalting showed a major protein band with good purity at 16 kD and a smaller, less intense band at 14 kD) (Figure 2). Further analysis of the purified and desalted SUMO-SA2 with size exclusion chromatography revealed two peaks (Figure 3, dashed line), of which the first peak eluted in the void volume of the column with a retention volume of 7.5 ml. The second peak eluted at 11.2 ml, with the same retention volume of a 14 kD globular protein (ribonuclease A). This indicated that SUMO-SA2 was present in monomeric as well as aggregated form. This can be explained by the hydrophobic nature of SA2 peptide causing the formation of aggregates. We assumed that the attachment of SUMO would prevent premature self-assembly of SA2 in bacteria, allowing us to purify the soluble fusion protein directly from the cleared lysates in a two-step metal affinity chromatography purification [6,20].

Here we showed that the presence of the SUMO fusion protein, although beneficial for expression levels of the SA2 peptide, did not completely prevent premature aggregation of the peptide-fusion construct.

### SUMO-SA2 cleavage

Next, SUMO-SA2 was cleaved with SUMO protease to release the SA2 peptide from the SUMO protein. SUMO protease was added to the SUMO-SA2 solution at a mass ratio of 1:500, and the mixture was incubated at 30°C for 6 hrs with gentle shaking. To monitor the enzymatic cleavage of SUMO-SA2, analytical size exclusion chromatography (SEC) was used (Figure 3, solid line). Before cleavage of SUMO-SA2, two peaks can be discerned, with the first peak eluting in the void volume of the SEC column and the second peak corresponding with monomeric SUMO-SA2. SDS-PAGE analysis confirmed that both peaks consisted of SUMO-SA2 only, suggesting that the first peak consists of a multimeric or aggregated form of SUMO-SA2. As expected, after cleavage a third peak at a retention volume of 15.9 ml appeared, which contained full length SA2 (as confirmed by mass spectrometry). Furthermore, after cleavage an increase in the area under the curve (AUC) of peak 1 and a decrease of AUC of peak 2 were observed, suggesting an increase in protein in the multimeric or aggregated form after cleavage.

**Figure 4:**
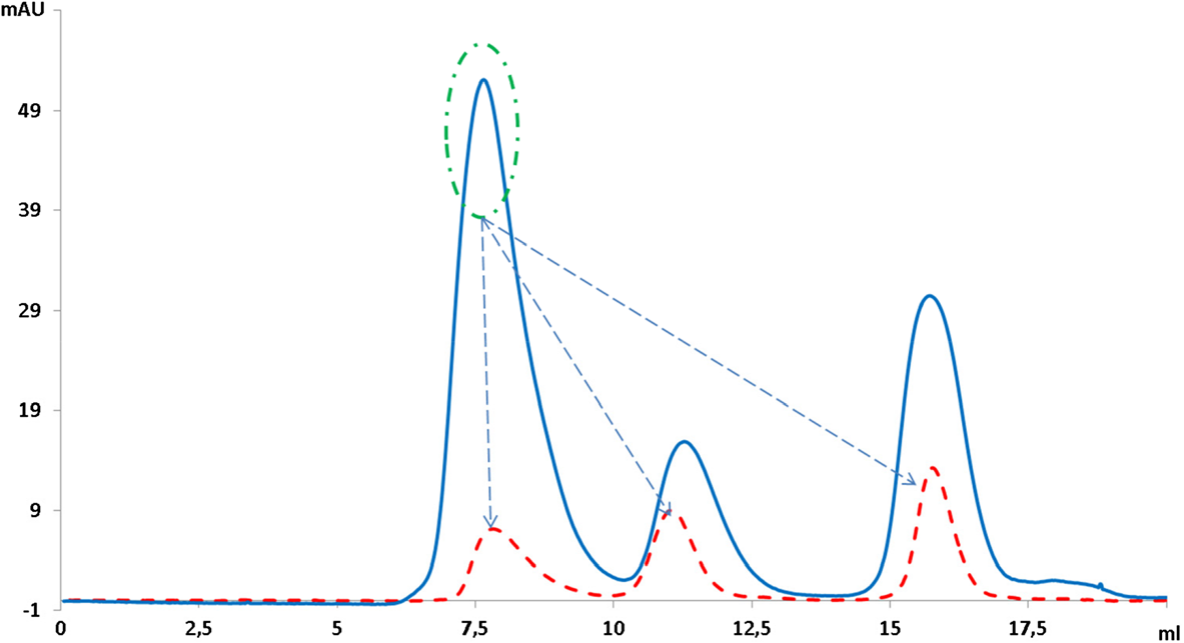
**Size exclusion chromatography analysis of cleaved SUMO-SA2 in phosphate buffered saline (pH = 7.4) Chromatogram showed three main peaks (solid line).** After collection and reinjection of the first peak the same three peaks appeared (dashed line). This indicates that the first peak contained complex aggregates of SUMO-SA2 and SA2.

To determine the composition of these aggregates, peak 1 was collected and again injected into the column. Interestingly, this fraction divided into three peaks, with the main part being monomeric SA2 peptide (Figure 4, dashed line). This demonstrated that the aggregate peak consisted of a mixture of SA2 and SUMO-SA2. In an attempt to dissolve the aggregates, we raised the pH of the sample and mobile phase to 11.5 prior to separation.

**Figure 5:**
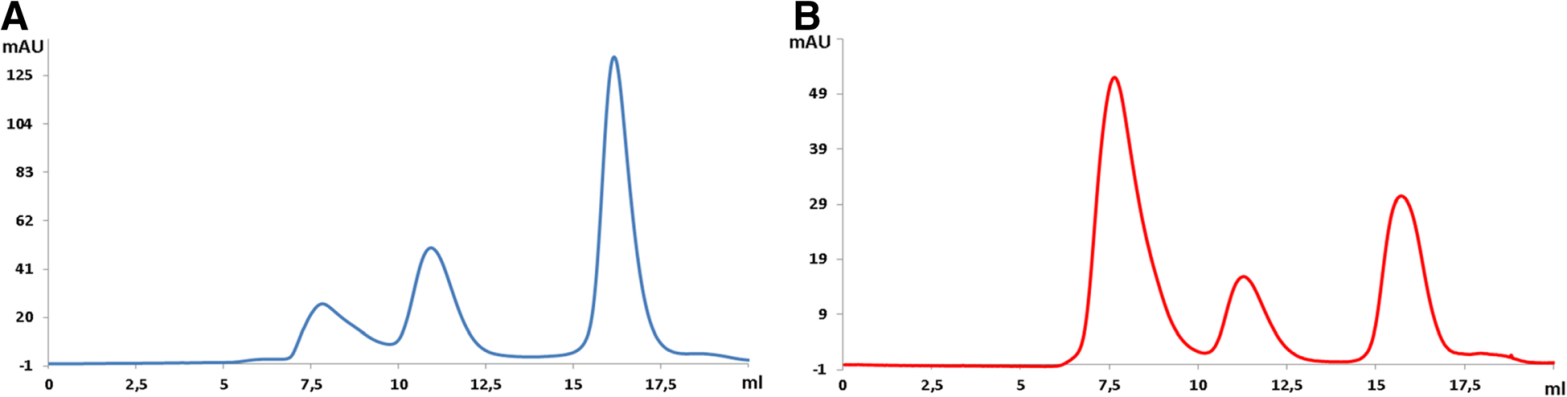
**The effect of alkaline medium on SA2 solubility.** Size exclusion chromatograms of the SUMO-SA2 protein solution after treatment by SUMO protease. Phosphate buffered saline pH = 11.5 **(A)** or phosphate buffered saline pH = 7.4 **(B)** were used as the mobile phase.

As shown in Figure 5A, SEC analysis was performed with phosphate buffered saline (pH 11.5) as the mobile phase. Interestingly, the AUC for the SA2 peptide peak clearly increased, while the AUC of the first peak simultaneously reduced, which showed that increasing the pH could dissolve the major part of the aggregates. This finding revealed that at high pH, the portion of SA2 peptides in a soluble, monomeric state was higher and consequently purification could be done more easily.

### Peptide purification

**Figure 6:**
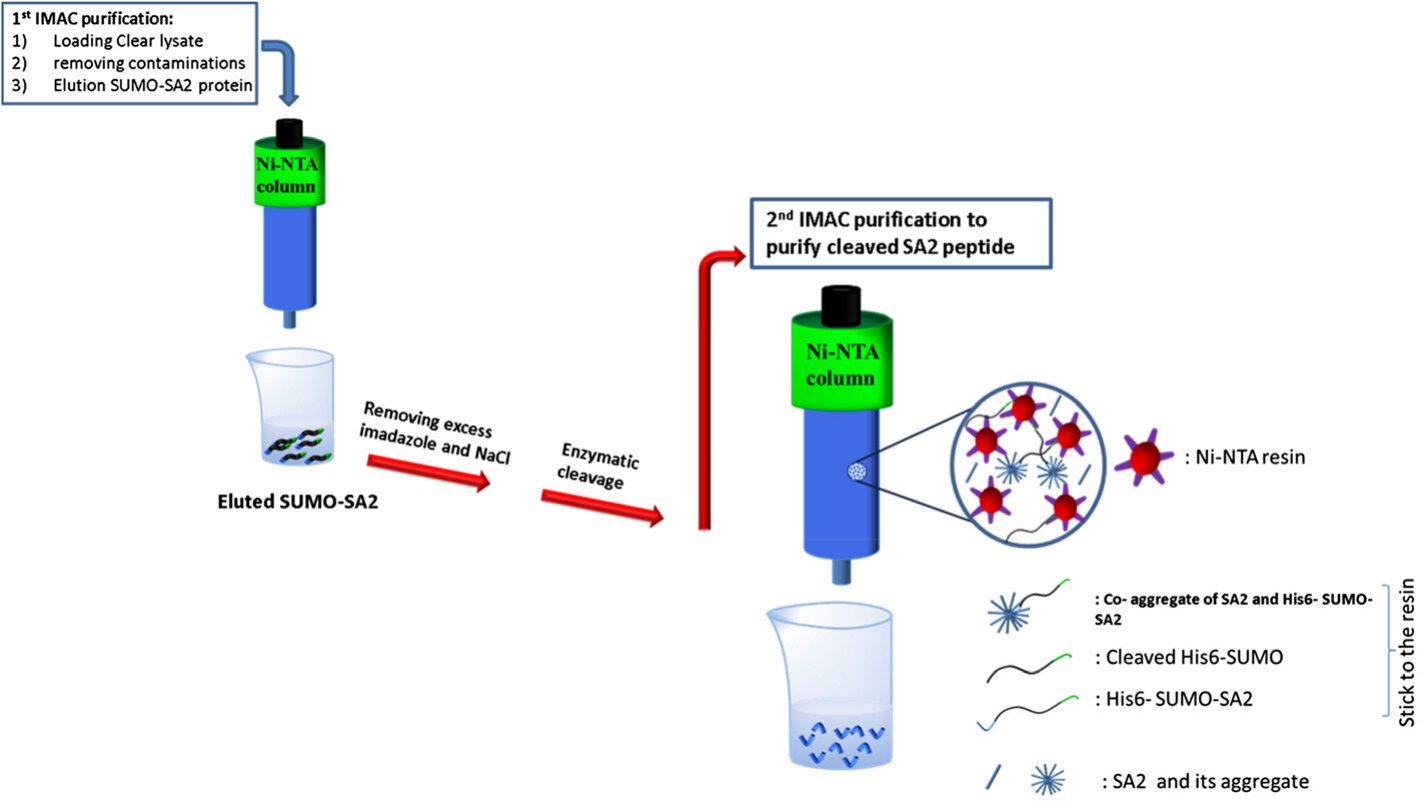
**Schematic representation of IMAC purification and the peptide loss in 2** ^**nd**^ **IMAC purification after cleavage of SUMO-SA2 by SUMO protease.** Released peptides are able to form aggregates with cleaved or uncleaved SUMO-SA2. Collected peptide in the flow through is low and most peptides stick to the column through the His tag of bigger proteins.

The original purification scheme for SUMO-SA2 entailed a two-step IMAC purification scheme in which the first step was the purification of SUMO-SA2 fusion protein from the cleared lysate. After cleavage of the purified SUMO-SA2 with SUMO protease, the His-tagged SUMO was separated from the released peptide by a second IMAC step. However, we observed that mixtures of uncleaved SUMO-SA2 and cleaved SA2 can form aggregates, which caused loss of the SA2 product during the second purification step (Figure 6). For that reason we adapted the purification scheme.

To separate the cleaved peptide from the SUMO protein, selective precipitation was applied. The pH of the protein solution after cleavage was adjusted to pH 11.5. SUMO, SUMO protease and uncleaved SUMO-SA2 were selectively precipitated by adding ethanol up to 50% (v/v) to the protein solution.

**Figure 7:**
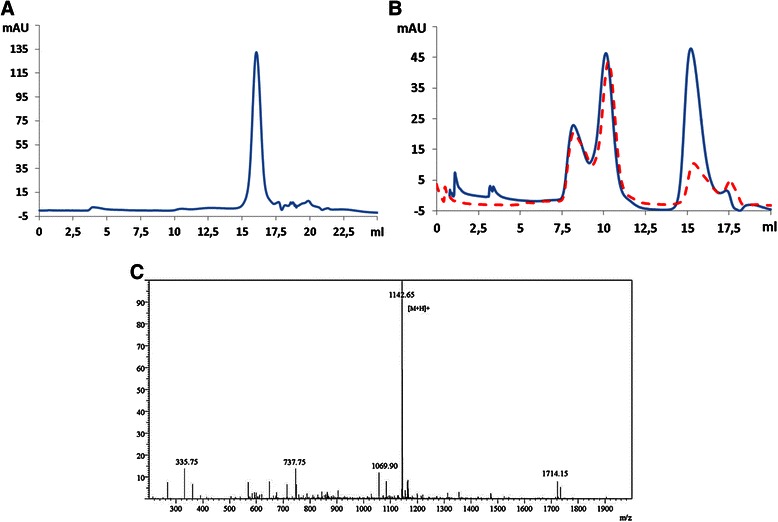
**Peptide purification after enzymatic cleavage through selective precipitation.** Selective precipitation was performed by adding ethanol up to 50% (v/v). A) Analysis of supernatant showed 1 major peak corresponding to SA2 **B)** SEC analysis of cleaved SUMO-SA2 before (solid line) and after (dashed line) selective precipitation. **C)** ESI-MS mass spectrum of the SA2 peptide in the supernatant after ethanol precipitation.

SEC analysis of the supernatant showed a major peak in the chromatogram, corresponding to the SA2 peptide which was confirmed by mass spectrometry (M.W. 1142.65 Da) (Figure 7A,C). To determine the yield of purification, the precipitate obtained after centrifugation was collected and resuspended in the same buffer and volume and analyzed by SEC. The chromatogram showed separation of most of the big proteins and aggregates and also some parts of SA2 (Figure 7B).

A comparison of the AUCs of the SA2 peptide peaks before and after ethanol precipitation revealed that 75% of the peptide was recovered in the supernatant (Table 1), and almost all other proteins were removed by ethanol precipitation.

## Conclusion

In conclusion, the results of this study demonstrated that premature self-assembly of SUMO-SA2 interfered with the proper purification of SA2 peptides resulting in low yields of purified peptide. By refining the purification procedure and by altering the expression medium, we demonstrated a more than 12-fold increase in purified SA2 peptide from one liter of bacterial culture. Although these findings are specific for the purification of SA2 peptides, premature self-assembly during recombinant production may also take place with other amphiphilic peptides, which may to a certain extent explain the low yields reported for the recombinant production and purification of such self-assembling peptides [21,26,27]. A critical evaluation of the purification scheme of such peptides may therefore be advisable.

## Materials and methods

### Materials

All chemicals and media were from Sigma-Aldrich (ST.Louis, USA), unless indicated otherwise. Bicinchoninic acid (BCA) assay reagent was from Pierce (Rockford, IL, U.S.A.). DNAse I was from Roche Diagnostics (Mannheim, Germany) and chicken egg white lysozyme was obtained from Fluka (Buchs, Switzerland; 84,468 U/mg). Hiprep 26/10 desalting and Superdex Peptide 10/300 columns were purchased from GE Healthcare, (Uppsala, Sweden). PageRuler™ Prestained Protein Ladder was from (Fermentas, Vilnius, Lithuania). Phosphate buffer saline (PBS) was obtained from Braun (Melsungen AG, Germany).

### Bacterial strains and plasmids

*Escherichia coli* BL21 (DE3) containing the T7 RNA polymerase under control of the *lacUV5* promoter was purchased from Invitrogen (Breda, The Netherlands). BL21 (DE3) was transformed with pET-SUMO-SA2 [6] and pSUPER-dtUD1 constructs (kindly donated by Prof. Patrick J. Loll) [28] separately according to the pET-SUMO supplier protocol (Invitrogen, Carlsbad, USA). Briefly, 6.5 ng of the plasmid DNA was added to 50 μl of chemically competent *E.coli* BL21 (DE3) in a Eppendorf tube and shaken gently. The tube was put on ice for 30 min after which the cells were placed in a water bath of 42°C for 30 sec. Next, the tube was placed on ice. For recovery of cells, 250 μl of SOC medium was added to the tube. To make a stock for the transformed *E.coli* bacteria, 100 μl of the bacterial suspension was transferred into a LB plate containing 50 μg/ml of kanamycin and incubated at 37°C overnight. A single colony was selected and grown in 5 ml LB overnight. The overnight grown bacteria were cooled on ice and glycerol was added up to 30% of final volume before storage at −80°C.

### Peptide biosynthesis

#### Media composition and protein expression

LB medium (peptone; 10 g/L, yeast extract; 5 g/L and 10 g/L of NaCl) was used for the pre-culture preparation and expression of SUMO-SA2 and SUMO protease (dtUD1). Auto induction medium (ZYM) was made according to the Studier method [24] and was used for SUMO-SA2 expression. In short, 1 L of ZYM medium that contained Tryptone (10 g/l), Yeast Extract (5 g/l), MgSO4 (1 mM), 20 mL of 50 × 5052 solution (glycerol 250 g/l, glucose 25 g/l, alpha lactose 100 g/l in RO water), kanamycin (100 mg/l) and 50 ml of 20× NPS solution ((NH_4_)_2_SO_4_ (66 g/l), KH_2_PO_4_ (136 g/l), Na_2_HPO_4_ (142 g/l). One liter of autoclaved ZYM or LB media was inoculated with 5 ml of overnight seed culture of the transformed *E. coli* strain BL21 (DE3). LB medium was incubated in a shaking incubator (Innova 4335, New Brunswick Scientifc,USA) at 37°C/250 rpm and induced with 1 mM IPTG when the culture reached OD_600_ = 0.6-0.8. Next, bacteria were harvested after 4 hrs by centrifugation at 5,000× g for 30 min at 4°C. Inoculated autoinduction medium was shaken at 37°C/250 rpm and bacteria were collected after 16 hrs at 5,000× g for 30 min at 4°C.

### Purification of SUMO-SA2

For the isolation and purification of the SA2 peptide, bacterial pellets were suspended in the lysis buffer (3 ml for each gram of biomass) (20 mM Na_2_HPO_4_, 150 mM NaCl, 20 mM imidazole, 5 mM MgCl_2_ 1.5% N-lauroylsarcosine, pH 8) supplemented with DNAse I 1 μg/ml and chicken egg white lysozyme 300 μg/ml. The resulted suspension was incubated on ice for 30 min. Subsequently, urea was added to the suspension to achieve 4 M final concentration. Lysis was accomplished using a Braun Labsonic tip-sonicator (Braun Biotech, Melsungen, Germany) for 5 min with 30 second stop between each 30 second pulse and passing two times through high pressure homogenizer. Next, the cell lysate was centrifuged (30 minutes, 40.000 g, 20°C) and supernatant was filtered through a 0.45 μm filter. SUMO-SA2 was purified by affinity chromatography using a 50 ml packed column of Ni-NTA Superflow (Qiagen, Chatsworth, CA) attached to an AKTA Purifier (GE Healthcare, Uppsala, Sweden). The column was washed with 5 column volumes of binding buffer (20 mM sodium phosphate, 0.5 M NaCl, 40 mM imidazole, pH 8) after which the cleared lysate was loaded onto a 50 ml packed Ni^2+^-NTA column at 0.5 ml/min at room temperature. After loading the cleared lysate, the column was washed with the binding buffer until the A_280_ reached to the baseline. His-tagged proteins were eluted from the column with elution buffer (20 mM sodium phosphate, 0.5 M NaCl, 500 mM imidazole, pH 8).

To remove excess imidazole and NaCl, the elution buffer was exchanged with cleavage buffer (20 mM hepes, 150 mM NaCl, pH 8.0) by loading onto a Hiprep 26/10 desalting column.

As the molecular weight of SA2 peptide (1.142 KD) is 8% of the molecular weight of the SUMO-SA2 (14295 KD), the highest expected amount of SA2 that can be released after enzymatic cleavage can be calculated.

### Purification of SUMO protease

The same protocol as described above was used for the purification of SUMO protease without addition of urea. Moreover, the elution buffer was replaced by the storage buffer (50 mM NaH_2_PO4, 300 mM NaCl, 1 mM DTT, pH 8.0) prior to protein quantification using the BCA assay which BSA used as a standard. Finally, 0.2 mg/ml dilutions were made by adding glycerol 50% (v/v) and stored at −80°C until required.

### Purification of SA2 peptide

SUMO protease was added at a 1:500 molar ratio to the purified SUMO-SA2 solution supplemented with 1 mM DTT and the mixture was incubated under gentle shaking for 6 hrs at 30°C to allow SUMO cleavage from the SA2 peptide.

To separate SA2 peptide from cleaved SUMO, SUMO protease and uncleaved SUMO-SA2, selective precipitation by ethanol was performed.

The pH of protein solution after cleavage was adjusted to 11.5 then ethanol was added up to 50% of the total volume to precipitate all proteins except SA2. After centrifugation at 5000xg at 4°C for 15 min, supernatant was collected and pH of supernatant was adjusted to 2 by adding 1 M HCl to precipitate SA2. The precipitate was collected and suspended in 0.1 M HCl and centrifuged at 5000× g at 4°C. This procedure was repeated 3 times.

Subsequently, recovered peptide was confirmed by HPLC, and mass spectrometry. Finally the peptide pellet was lyophilized at −50°C and at 0.5 mbar in a Chris Alpha 1–2 freeze-drier (Osterode am Harz, Germany) for 12 hrs and stored at −20°C.

### Characterization of produced peptide

#### Gel electrophoresis

The produced proteins were evaluated by SDS-PAGE. Samples were boiled in Laemmli sample buffer (Bio-Rad Laboratories, Hercules, CA, USA) for 5 min and loaded at 20 μl/well onto NuPAGE 10% Novex Bis-Tris gels (12 wells, 1.0-mm thickness; NuPAGE, Invitrogen, Carlsbad, CA, USA). Electrophoresis was performed at room temperature applying a constant voltage of 175 V for 50 min. The gel was stained with Page Blue™ Protein Staining Solution (Fermentas GMBH, St. Leon-Rot, Germany) and destained overnight by washing with RO water.

### Size exclusion chromatography

Cleavage of SUMO-SA2 was followed by Size Exclusion Chromatography on a Superdex Peptide 10/300 GL column at a flow rate of 0.7 ml/min with phosphate buffered saline at pH = 7.4 or pH = 11.5 as the mobile phase. Prior to loading the samples a Gel Filtration LMW Calibration kit (GE Healthcare, Uppsala, Sweden) was used to validate column performance.

### HPLC analysis and mass spectrometry

1 mg of Lyophilized SA2 peptide was dissolved in 1 ml of DMSO and 20 μl of the peptide solution was diluted 5 times in RO water. 50 μl of prepared sample was injected onto a Sunfire C18 column (waters Corporation, Milford, USA). A gradient was run at 1.0 ml/min flow rate from buffer A (5% actonitrile, 0.1% trifluoroacetic acid, 95% water) in 30 minutes to buffer B (100% acetonitrile, 0.1% trifluoroacetic acid). UV absorption was monitored at 220 nm, 280 nm and also fluorescent emission at 350 nm of tryptophan residue upon excitation at 295 nm was recorded.

Furthermore, Electrospray ionization (ESI) mass spectrometry was carried out using a Shimadzu LCMS QP-8000(Duisburg, Germany) single quadrupole bench top mass spectrometer (m/z range, 2000), coupled with a QP-8000 data system.

## Abbreviations

A280: Absorbance at 280 nm
Aps: Amphiphilic peptides
AUC: Area under the curve
BSA: Bovine serum albumin
DMSO: Dimethylsulfoxide
DNase: Deoxyribonuclease
DTT: Dithiothreitol
E.coli: Escherichia coli
G: Relative centrifugation force to gravity
HEPES: 4-(2-hydroxyethyl)-1-piperazineethanesulfonic acid
His6: Hexa-histidine tag
HPLC: High-performance liquid chromatography
IMAC: Immobilized-metal affinity chromatography
IPTG: Isopropyl-β-D-thiogalactopyranoside
LB-medium: Luria-Bertani medium
Ni-NTA: Nickel-nitriloacetic acid
OD600: Optical density at 600 nm
RNase: Ribonuclease
RO water: Reverse osmosis water
Rpm: Round per minute
SDS-PAGE: Sodium dodecyl sulphate-polyacrylamide gel electrophoresis
SEC: Size exclusion chormatography
SOC: Medium super optimal broth medium + glucose
SUMO: Small ubiquitin-like modifier
